# Letter from Dr. E. J. B. Ft. Worth

**Published:** 1886-11

**Authors:** 


### "TAIT'S OPERATION."

FORT WORTH, Texas, November 14, 1886.

*Editor Daniel's Texas Medical Journal:*

What do our Texas journals and surgeons mean by Tait's operation?

Mastin has said that the removal of the ovaries should bear the name of Battey. The removal of the tubes the name of Tait. The removal of both, ovaries and tubes, the name of Hagar. Tait has expressed the wish that his name be attached to the removal of the uterine adnexa for *bleeding uterine myoma.*

What do Wilkinson and others mean by Tait's operation? I have made nine laparatomies, or as Tait prefers, nine abdominal sections. Yours, etc. E. J. B., M. D.

